# Research priorities for mental health and circadian science: a priority setting partnership of individuals with lived experience, carers, clinicians and researchers

**DOI:** 10.1136/bmjment-2025-302101

**Published:** 2026-02-09

**Authors:** Amy C Ferguson, Ivana Kamenska, Nahid Ahmad, Nicole Needham, Michael Farquhar, Candida Stephens, Usayd Abid, Dylan Perry, Maria Gardani, Nick Meyer, Haya Deeb, Katie F M Marwick, Daniel J Smith, Malcolm von Schantz, Alice M Gregory

**Affiliations:** 1 Institute for Neuroscience and Cardiovascular Research, University of Edinburgh, Edinburgh, UK; 2 Warwick Medical School, University of Warwick, Coventry, UK; 3 Evelina London Children’s Hospital, London, UK; 4 The McPin Foundation, London, UK; 5 Insomnia and Behavioural Sleep Medicine Clinic, University College London Hospitals NHS Foundation Trust, London, UK; 6 School of Biological Sciences, University of Edinburgh, Edinburgh, UK; 7 Faculty of Health and Life Sciences, Northumbria University, Newcastle upon Tyne, UK; 8 Department of Psychology, Royal Holloway University of London, Egham, UK

**Keywords:** Psychiatry, Mental Health

## Abstract

**Background:**

Undisturbed circadian rhythms of rest/activity are crucial to health and well-being. There is growing evidence to suggest that circadian rhythm disruptions are also associated with adverse mental health outcomes (and vice versa), but important questions about the relationship between circadian rhythms and mental health remain unanswered.

**Objective:**

To determine future priorities for research in the area of mental health and circadian rhythms, a James Lind Alliance Priority Setting Partnership exercise in collaboration with a steering group comprising individuals with lived experience, carers and clinicians was undertaken.

**Methods:**

An initial survey among UK residents provided a set of 964 questions supplied by 247 respondents (227 lived experience, 44 carers (including 40 carers with lived experience), 41 clinicians (including 37 clinicians with lived experience)). Responses were processed into 171 summary questions by the steering group. Reviews of published research and existing clinical guidelines reduced this to 63 unanswered summary questions. A ranking survey of these 63 questions asked respondents to select their 10 most important research questions, from which the most highly ranked would be taken to the final stage. This was completed by 222 respondents (200 lived experience, 33 carers (including 29 carers with lived experience), 38 clinicians (including 30 clinicians with lived experience)).

**Findings:**

In a final face-to-face workshop, 19 individuals, including individuals with lived experience, carers and clinicians, discussed and ranked a list of questions to produce a ranking of the top 25 research questions/priorities, with a particular focus on the Top 10.

**Discussion:**

The final research questions are presented to inform researchers and funding bodies when setting future research priorities across the fields of mental health and circadian rhythms.

**Clinical implications:**

Addressing the priorities identified here should lead to greater understanding of the relationships between mental health and circadian rhythms and will have longer-term impacts on research, healthcare innovation and public health policy.

**WHAT IS ALREADY KNOWN ON THIS TOPIC**

- There is growing evidence of the relationships between circadian rhythms and mental health. However, many important questions remain unanswered.

**WHAT THIS STUDY ADDS**

- This study is the first of its kind in the area of mental health and circadian science. Here, we identify the research priorities of individuals with lived experience of mental health difficulties and/or circadian rhythm disruption, carers and clinicians involved in supporting them.

**HOW THIS STUDY MIGHT AFFECT RESEARCH, PRACTICE OR POLICY**

- This process will direct the research agenda to address questions that are important to people with lived experience, and may also help deliver information and innovation that is relevant and valued by people with lived experience of circadian disruption and poor mental health.

## Introduction/background

 Endogenous 24-hour (circadian) rhythms are found across almost all forms of life, from unicellular organisms to humans.^1^ Expressed at multiple physiological and behavioural levels, circadian oscillations allow organisms to optimally align physiology and organise behaviour to predict, as opposed to solely reacting to, the daily cycles of light and darkness.^1^ Appropriate alignment between circadian pacemakers and the day–night cycle, and synchrony between oscillators within an organism, is fundamental for human health and is important for mental wellbeing.^2 3^ However, many factors, including patterns of modern living (such as shift work and artificial light at night), can cause misalignment and desynchronisation of rhythms, resulting in a wide range of adverse mental health outcomes.^4^,^7^ This may be particularly important for young people, who may be more sensitive to light-induced circadian dysfunction and associated mental health problems.^8 9^

Even though mental health disorders such as depression, bipolar disorder and psychosis are often associated with aberrant circadian rhythms and subsequent effects on sleep/wake cycles,^10 11^ the mechanisms of these associations remain poorly understood. The clinical application of the discoveries made in chronobiology over recent years within mental health services has so far been limited.^12^,^14^ More generally, the fundamental importance of healthy rhythms of sleep has historically been overlooked within public mental health initiatives. As is the case in many other areas of research, the voices of people with lived experience, as well as those working with them (carers and clinicians), have not been adequately represented in previous research in this area.

There is, consequently, a need to identify and prioritise the most important research questions at the interface between circadian rhythms and mental ill health, to ensure that future research and innovation is strategic and considers the views of people with lived experience, carers and clinicians. In general, the priorities for research are directed by researchers, funding bodies, invested industries and policymakers. Those who experience the conditions studied, as well as those who care for and treat them, are often provided little to no opportunity to input into these priorities. The importance of incorporating the voices of lived experience into research to ensure outcomes are as beneficial as possible to those most impacted by the conditions is, rightly, becoming more widely recognised.^15^,^17^ The James Lind Alliance (JLA) provides gold-standard, structured methodology for conducting priority setting partnerships (PSPs) that bring together patients, carers and healthcare professionals to set a list of priorities for research in areas of healthcare.^18^ Here, we describe the process and outcome of the first PSP to be conducted on the topic of ‘the body clock and mental health’ to identify future priorities for research selected by individuals with lived experience of mental health difficulties and circadian disruption and those who support them.

### Objective

The aim of this PSP was to generate the first list of research questions on mental health and circadian rhythms considered to be the most important for individuals with lived experience, carers and clinicians with the ambition to influence the direction of future research and innovation.

### Clinical implications

The PSP has identified and prioritised a diverse range of research questions. Addressing these should lead to greater understanding of the associations between circadian rhythms and mental health, as well as longer-term impacts on research, healthcare innovation, clinical practice and public health policy.

## Methods

**Figure 1:**
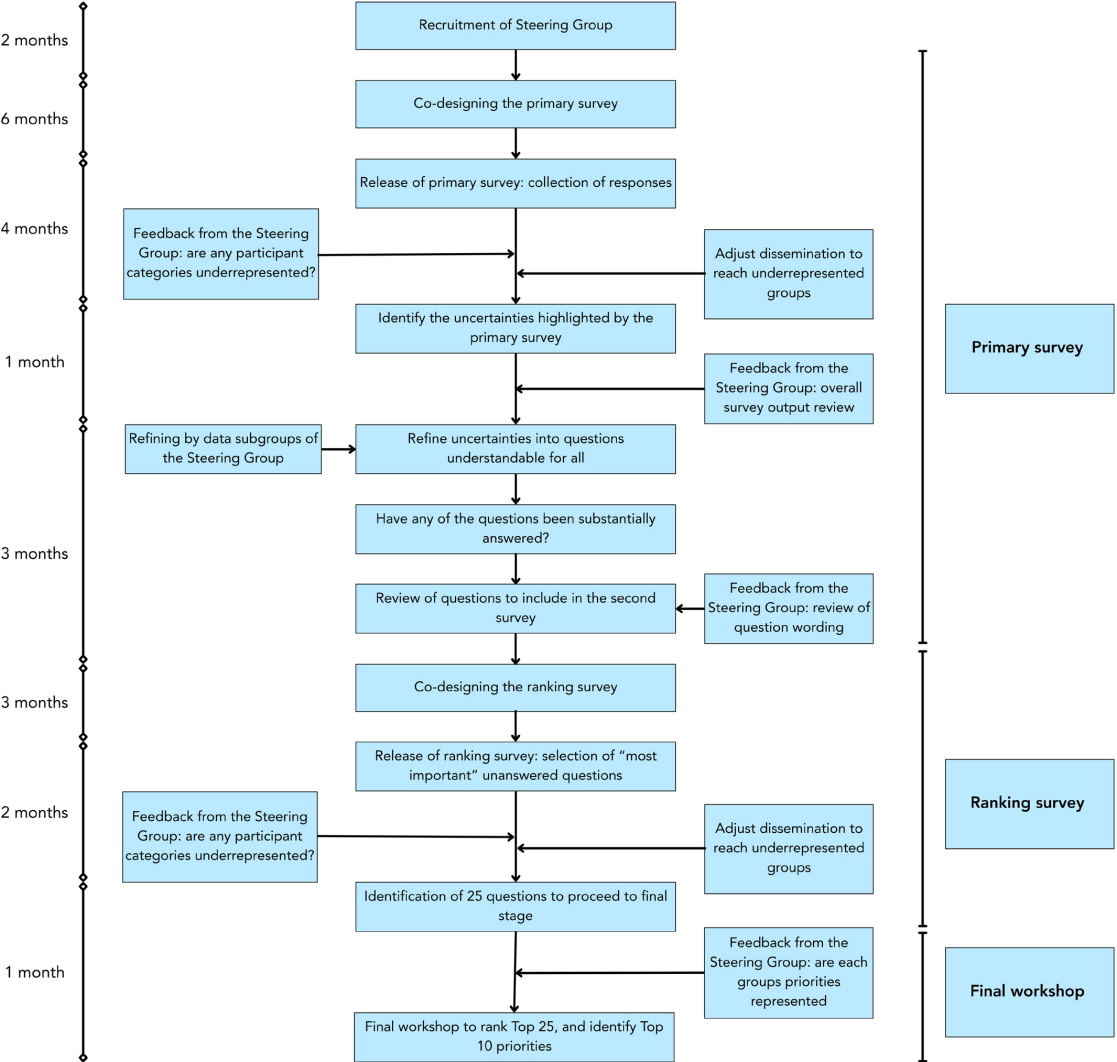
Outline of priority setting partnership process.

This PSP adhered to the well-established JLA guidelines to undertake a priority setting exercise (figure 1).^19^ The first step was to recruit a steering group to input into each of the following steps within the PSP and work alongside the research team (ACF, IK, MvS, AMG). To recruit a steering group, the team reached out directly to potential stakeholders who would be interested in the priorities of future research in mental health and circadian science. This included (but was not limited to) The McPin Foundation, The Sleep Charity, Mental Elf, Bipolar Scotland and clinicians working across circadian rhythm disruption and mental health. An open invitation to express interest in joining the steering group was also sent out via the Circadian Mental Health Network mailing list and social media platforms.

The steering group was composed of 14 individuals: 9 individuals with lived experience of mental health difficulties and/or circadian rhythm disruption, 5 individuals who care for/support individuals with these lived experiences and 6 clinicians who work within the fields of mental health, sleep and circadian rhythms (including NN, MG, NM, MF, CS, UA, DP who accepted an invitation shared with the whole steering group to become authors of the manuscript). Some of the individuals with lived experience are members of the Circadian Mental Health Network’s McPin Foundation Lived Experience Advisory Panel. Some members of the steering group fell into multiple categories.

The PSP chose the name ‘Mental Health and the Body Clock’ to facilitate clear understanding of the term ‘circadian rhythms’ (even though the term ‘body clock’ covers other body clocks such as ultradian or circannual rhythms), this was agreed on by the steering group. At the beginning of each survey, the term body clock was described as another term for circadian rhythms with a definition which aligns with published definitions of circadian rhythms.^1^ The explanation from the participant information read as follows: ‘Body clocks are internal rhythms that are essential to physical and mental functions. These internal clocks help to prepare our body and mind to best align with our environment. Body clocks also react to our environment and can change when our environment does. For example, they can prepare our bodies and minds for when we should eat, be active, be alert, rest, sleep, wake up… and so much more.’ A decision was taken at the very beginning of the PSP to focus on mental health and circadian rhythms (the body clock), and to exclude the word ‘sleep’ from the remit. This reflects the specific research interests of the Circadian Mental Health Network which focuses on circadian rhythms of all types, of which sleep is only one example. Despite this PSP being led by the Circadian Mental Health Network research team, the researchers had no influence on the selection or prioritisation of the research priorities, in accordance with JLA principles. The PSP protocol was agreed by the steering group and published on the JLA website (https://www.jla.nihr.ac.uk/documents/mental-health-and-body-clock-psp-protocol).^20^

Each survey was co-designed with the research team and the PSP steering group. The study materials, surveys and promotional materials were co-designed and reviewed by the steering group (all survey materials can be found in the online supplemental material). This ensured that there was input from those with lived experience, clinical and care experience at each stage of the PSP (figure 1). At crucial stages of the PSP process, the steering group were approached for feedback of the process itself (eg, before the launch of each survey they were asked ‘Do you have any comments that you have not been yet been able to make?’, and after data analysis asked ‘How did you find this process and is there anything we could do to improve this?’), including through open text feedback surveys to encourage any anonymous comments or suggestions for the research team to ensure they found the coproduction effective.

### Participants

PSP activities, including both surveys and the workshop, were only open to UK residents over the age of 11. The project was based in the UK, and so the team were limited in their capacity to address questions that would be specific to the societal and cultural contexts outside of the UK. Based on recommendations from steering group members who work with children, age 11 was determined to be a reasonable age of assent to take part in the project. Both published surveys and all public engagement materials were reviewed. Each survey contained an information sheet and asked respondents to confirm that they had read the information and wished to take part in the survey.

### Dissemination

To disseminate the surveys, various communication strategies were used. Both surveys were posted on the Circadian Mental Health Network website and via the Circadian Mental Health Network social media accounts. This included the Network’s mailing list, comprised of individuals with lived experience, carers, clinicians and researchers. The surveys were distributed by the steering group members via both professional and personal networks. The team worked with partners including The McPin Foundation (an organisation focused on supporting the voices of those with lived experience), Bipolar Scotland and The Sleep Charity to publicise the survey via events, blogs, newsletters and social media channels.

### Primary survey

The steering group co-designed the primary survey with the researchers leading the project. The decision was made to include four open-ended questions, designed to encourage respondents to give short text answers and to avoid directing respondents, but no character limit was placed on the possible responses. The questions covered in the primary survey were: ‘What would you like to know about how a disrupted body clock impacts on mental health?’; ‘What would you like to know about how mental health difficulties impacts the body clock (eg, issues staying awake/alert)?’; ‘Do you have any questions about Mental Health and the Body Clock that you feel research needs to answer?’ and ‘Do you have any further thoughts or experiences about Mental Health and the Body Clock you would like to share?’ (online supplemental figure 1A).

Respondents were also asked some sociodemographic questions. The survey was available online via Microsoft Forms, and we also produced a paper version of the survey. The primary survey was disseminated using the strategy detailed above.

### Refining the questions

All responses were assessed by ACF and IK to identify responses which should be excluded based on prespecified criteria. We excluded responses that were out-of-scope as designated by the PSP protocol (https://www.jla.nihr.ac.uk/documents/mental-health-and-body-clock-psp-protocol)^20^ including questions referring to specific geographical locations, questions about sleep disorders with a non-circadian cause (eg, obstructive sleep apnoea), questions asking about specific current policies and responses that were too broad to extract a specific question. All excluded questions were reviewed and agreed on by the steering group (figure 1). All remaining responses, asking any question referring to circadian rhythms and mental health, that were deemed in-scope (as per the protocol) were collated into various groups, producing 69 themes (online supplemental table 1) and similar questions within those themes were merged to create potential questions for the ranking survey. Data subgroups, comprising steering group members, reviewed this process to ensure questions chosen for merging were appropriate (figure 1). They also revised the language used to maximise accessibility. For example, terms such as ‘cognitive function’ were expanded for clarity, and the use of person-centred language (eg, ‘individuals with insomnia’) replaced condition-first language (eg, ‘insomniac’). To identify summary questions which were not substantially answered by previous research, a traffic light system was used. Red indicated questions which were not substantially answered, yellow indicated partially answered questions and green was used for questions with a research focus evident in peer-reviewed literature or for questions which had been substantially answered and were, therefore, excluded (addressed by systematic review, meta-analysis and/or current clinical guidelines as indicated in the JLA guidelines). This process was reviewed by steering group members and additional clinicians (including HD, KFMM) (figure 1).

### Ranking survey

The aim of the ranking survey was for respondents to choose the 10 questions that were most important to them, from the questions formulated from the primary survey (figure 1). It was decided by the steering group that it would be too burdensome to ask respondents to rank their top 10 questions out of 63 available questions at once. This second survey therefore comprised two parts: (1) respondents were asked to select all questions they felt were important for research to answer; (2) respondents were then asked to select the 10 most important to them from those selected in step 1. The survey contained the same optional sociodemographic questions as the primary survey.

Additional descriptions for terms such as ‘neurodivergent’ and ‘postpartum depression’ were included for the relevant questions within this survey. This was a request from the steering group to maximise the accessibility and understanding of the survey. Survey 2 was created using Qualtrics (a PDF version was also created for respondents who required a printable version (online supplemental figure 1B)).

### Identification of questions for final prioritisation

We initially selected the 15 most frequently selected questions in stage 2 of the second survey from each group: individuals with lived experience, carers and clinicians. Due to overlap in choice between the three groups, this resulted in 28 unique questions. These 28 questions were taken forward to the steering group for review and selection of 25 questions to be taken to the workshop stage (figure 1). To select these 25, an emphasis was placed on how frequently each question was selected by the lived experience group as they were of a high priority to this PSP process. With the final selected 25 questions, we had complete coverage of the ‘top’ 14 for those with lived experience, the ‘top’ 6 for clinicians and ‘top’ 4 for carers (with ‘top’ referring to the most frequently selected questions).

### Confirmation of the final priorities

Participants of the ranking survey were invited to register their interest to attend the final workshop at the end of the ranking survey, and those individuals with lived experience, carers or clinicians who expressed an interest were invited to join the final workshop. Invitations to express interest were also sent out via the Circadian Mental Health Network mailing list and directly to clinicians working within the fields of mental health and circadian rhythms. Those who replied to these invitations were asked to confirm their experience and/or clinical background and to confirm there were no potential conflicts of interest. The number of responses we received did not outweigh the available workshop spaces, so no eligible participant wanting to take part was excluded. The workshop was attended by 19 participants, some of whom represented multiple categories of experience, with 11 individuals with lived experience, 2 carers and 9 clinicians. Of these participants, 6 were also members of the steering group.

Ahead of the workshop, participants were asked to rank the 25 research questions shortlisted by the steering group to allow expression of their personal preference and to ensure familiarity with the questions. The workshop was facilitated by two JLA advisors and observed by four individuals (one JLA advisor in training and three researchers from the Circadian Mental Health Network) who did not express an opinion on the questions during discussions and did not have any influence on the ranking of priorities. The workshop attendees addressed the questions both as a single large group and were then split into two smaller groups. The smaller groups were preselected to ensure a balance of individuals with lived experience, carers and clinical representation.

The workshop involved five exercises: (1) the small groups were asked to discuss their highest and lowest priorities from their assessment of the questions before the workshop, (2) the small groups were asked to rank all 25 questions, (3) the rankings of the two small groups were combined and discussed as one large group, (4) the entire group was mixed and divided into two different small groups (again ensuring balance of roles), to ensure different views were heard and asked to assess the combined ranking and make any changes to this through discussion and (5) the new rankings for the two groups were combined and discussed in the large group.

## Findings

**Figure 2:**
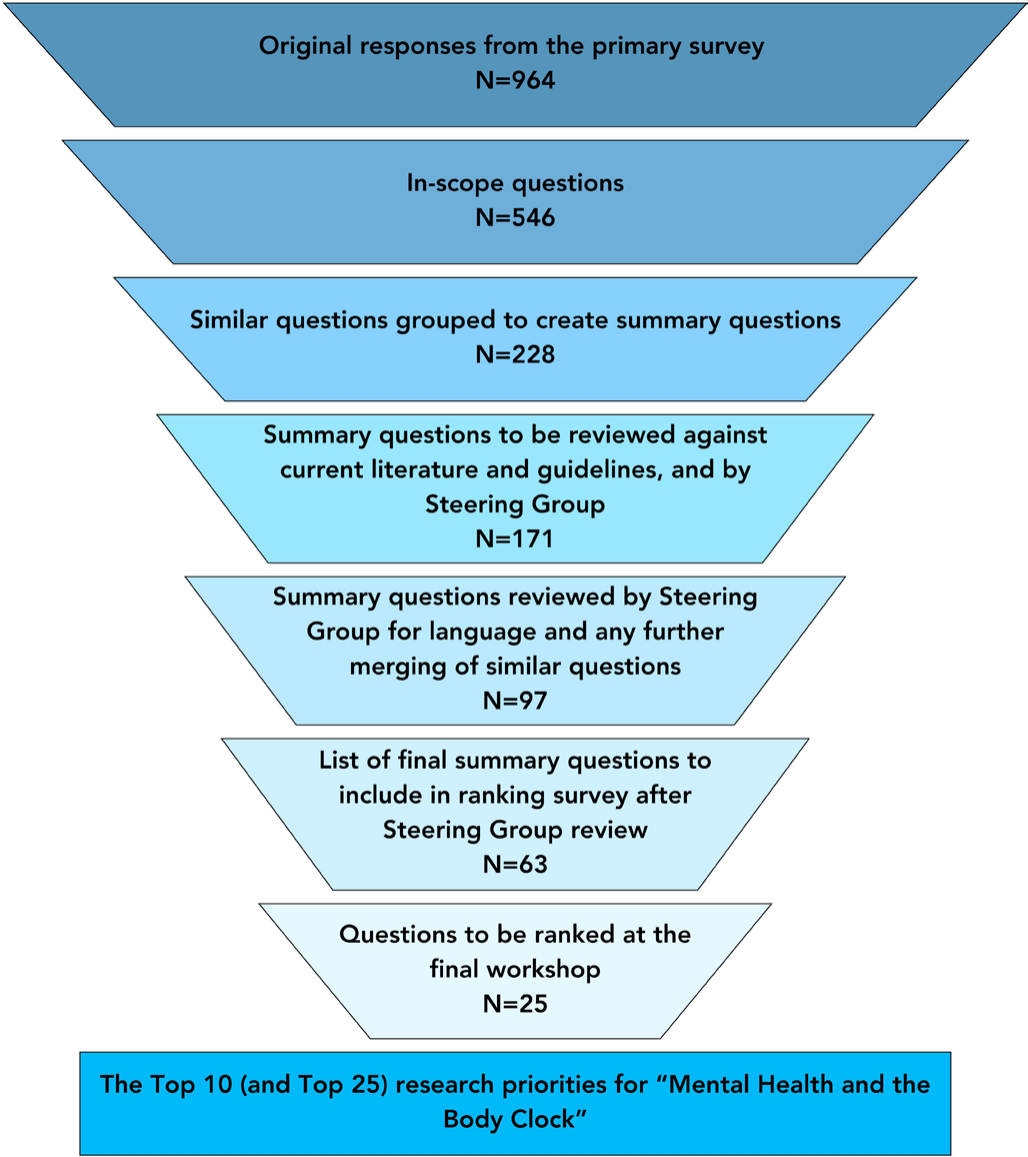
Priority setting partnership (PSP) process of identifying, summarising and selecting research priorities.

A total of 274 participants took part in the initial survey (with 3 taking part via paper survey), 227 with lived experience, 44 carers (including 40 carers with lived experience), 41 clinicians (including 37 clinicians with lived experience) and demographic information is provided in table 1. There were 27 participants excluded as they were not UK residents. This resulted in 964 question submissions, 418 of which were excluded as they were considered out-of-scope or too vague (such as references to specific geographical locations, responses that a specific question could not be interpreted from), to provide 546 questions (figure 2).

**Table 1:** Demographic information of respondents from each survey

	Primary survey, n=247	Ranking survey, n=222
Age (years)		
Mean (SD)	43.6 (15.2)	43.2 (14.2)
Range (min–max)	12–95	12–82
Gender		
Female	171 (69.2%)	121 (54.5%)
Male	59 (23.9%)	47 (21.2%)
Other	9 (3.6%)	10 (4.5%)
Gender not stated	8 (3.2%)	44 (19.8%)
Experience (not mutually exclusive)		
Mental health difficulties or a mental health condition	159 (64.3%)	143 (64.4%)
Disrupted body clocks (caused by shift work, jet lag, lifestyle)	129 (52.2%)	70 (31.5%)
Disrupted body clocks (such as sleep disturbances, sleeping when should be awake and/or disruptions of daily routine/activities)	169 (68.4%)	121 (54.5%)
Supporting individuals with mental health conditions or disrupted body clocks (family, friend, carer, counsellor, clinician, clinical support staff of people with experience of mental health conditions or disrupted body clocks)	92 (37.2%)	104 (46.8%)
Experience not stated	6 (2.4%)	8 (3.6%)
Group (not mutually exclusive)		
Individuals with lived experience	227 (91.9%)	200 (90.1%)
Carers	44 (17.8%)	33 (13.4%)
Clinicians	41 (16.6%)	38 (15.4%)
Location		
Scotland	80 (32.4%)	59 (26.6%)
England	152 (61.5%)	115 (51.8%)
Wales	3 (1.2%)	1 (0.5%)
Northern Ireland	1 (0.4%)	3 (1.4%)
Location not stated	11 (4.5%)	44 (19.8%)
Ethnicity		
White British or white other	212 (85.8%)	159 (71.6%)
Black British, black, African or Caribbean	4 (1.6%)	3 (1.4%)
Asian British or Asian other	13 (5.3%)	16 (7.2%)
Hispanic or Latina/o/x	2 (0.8%)	2 (0.9%)
Other	1 (0.4%)	4 (1.8%)
Mixed	5 (2.0%)	9 (4.1%)
Ethnicity not stated	10 (4.0%)	29 (13.1%)

These raw submissions were then categorised into 69 different themes reflecting the broad scope of this PSP (online supplemental table 1). The raw submissions were assessed for similarity and combined into 171 summary questions, through extensive review by the steering group (figure 2).

There were 74 questions considered substantially answered on review by the research team and steering group. The remaining 97 questions went through a further assessment of similarity, combining (including the decision to combine similar questions about specific mental health conditions into single questions addressing ‘mental health conditions’) and rephrasing to create 63 summary questions to populate a second survey (figure 2).

The ranking survey received 320 responses (2 of which were submitted on printed versions of the questionnaire), 92 responses were excluded due to non-completion of the survey and 6 were removed as they were completed by non-UK residents. The ranking survey was completed by 200 individuals with lived experience, 33 carers (including 29 with lived experience) and 38 clinicians (including 30 with lived experience). The steering group shortlisted 25 questions from the most frequently selected questions from individuals with lived experience, carers and clinicians (figure 2, online supplemental table 2), which included a diverse range of questions including those relating to mechanisms, the impact of environmental factors and the relationship between mental health and circadian rhythms in different conditions.

The workshop resulted in full consensus around the Top 10 priorities for ‘Mental Health and the Body Clock’. The ranked priorities did show a marked difference compared with the rankings from the second survey. This is likely due to the nature of the final workshop which produces a group consensus of the workshop attendees as opposed to the individual views from the ranking survey. Table 2 details the Top 10 priorities for research in mental health and circadian science (the rankings of all 25 questions are found in online supplemental table 2).

**Table 2:** The Top 10 priorities for research in Mental Health and the Body Clock

Ranking in final Top 10	Question	Ranking from second survey by		
		People with lived experience	Carers	Clinicians or clinical support workers
1	Does the interaction between Mental Health and the Body Clock vary by age, especially during different life stages?	15th	1st	2nd
2	What strategies (including medications) are effective in treating disrupted body clocks co-occurring with mental health issues?	13th	5th	6th
3	What is the relationship between the body clock and mental health in neurodivergent individuals and does body clock disruption worsen mental health in these individuals?	8th	3rd	5th
4	What is the relationship between a disrupted body clock and bipolar disorder, or between a disrupted body clock and psychosis? What are the mechanisms involved in this?	11th	8th	4th
5	What societal and/or policy changes can help prevent mental health issues for, and reduce stigma towards, extreme chronotypes?	13th	8th	8th
6	What is the relationship between (peri)menopause, mental health and body clocks?	2nd	2nd	1st
7	How does mental trauma (eg, grief) affect the body clock? How can this be managed?	9th	7th	8th
8	Would it be better for a person’s mental health to follow their own (natural) rhythms or to follow more typical sleep patterns and/or social patterns?	4th	9th	6th
9	What is the relationship between seasonal changes, body clocks, mental well-being and mental health issues?	6th	4th	7th
10	Can mental health difficulties, such as anxiety or depression, cause disruption of the body clock at a molecular level or are these driven mainly by behavioural factors?	13th	6th	4th

The ranking by those with lived experience, carers and clinicians comes from the second survey (these rankings informed the steering group selection of these questions for the workshop stage). The individual rankings from the different groups do therefore not directly map onto the Top 10 research priorities which were agreed at the final workshop.

### Assessment of the coproduction process by individuals with lived experience

The objective of this PSP was to identify the priorities for research in mental health and circadian science, ensuring the involvement of individuals with lived experience throughout. Partnering with lived experience organisations such as The McPin Foundation allowed those voices to be incorporated into each stage of the PSP. As a major objective of the Circadian Mental Health Network, it was crucial to coproduce and co-design the surveys, data analysis and public materials. The success of this process was reflected in the feedback from the steering group members with lived experience, which was obtained through requesting feedback on completion of the project. Quotes include:

Learning about both common concerns and everyone’s diverse perspectives regarding the body clock and sleep was very enriching.

My role typically involved reviewing, evaluating and refining the research questions that emerged from data collection and wider consultation. I contributed to achieving a fair representation of questions across the different themes, whilst also supporting the fair prioritisation of those selected for the final list.

From the start it felt as if this was true co-production and co-design. In our discussions of how to formulate the survey questions it seemed so appropriate to solicit the thoughts of exactly the people who would be answering the survey questions and who might not understand the technical terms that are second nature to the scientists and researchers.

The feedback received was very positive about the process that was used throughout this PSP to ensure the inclusion of individuals with lived experience of mental health difficulties and circadian disruption.

## Discussion

The aim of this PSP was to identify the future priorities for research in mental health and circadian science, as directed by those with lived experience of mental health and circadian rhythm disturbances and those who care for and treat them. This PSP is the first of its kind in this area. Through this PSP, 25 questions have been identified as priorities for research, with a focus on those ranked as the Top 10. These priorities will influence future research and innovation.

The Top 10 research priorities can be grouped into four broad categories (with some of the questions belonging to more than one). Six of the questions are mechanistic in nature (questions 3, 4, 6, 7, 9 and 10 in table 2), in one case (question 4) explicitly so. Three questions were concerned with clinical management or self-management (questions 2, 7, 8). Seven questions propose topics for observational and/or longitudinal studies (questions 1, 3, 6, 7, 8, 9, 10). Overall, the majority of the Top 10 questions related to basic research rather than clinical intervention, reflecting a primary desire for improved understanding of circadian and mental health dysfunction among stakeholders. One question (question 5) directly asks for evidence for potential public policy interventions.

Questions 3, 4, 6, 7, 9 and 10 (table 2) are mechanistic in nature and can be expressed as clear directional hypotheses. Overall, their prioritisation highlights how the exact pathways which influence mental health and vice versa are not well understood. Some research already addresses the relationships between circadian rhythms and mental health conditions and neurodiversity, for example, in bipolar disorder,^21^ attention deficit and hyperactivity disorder^22^ and autism,^23^ but this is incomplete and limited to specific conditions. Extensive work has also been done to detail the underlying molecular basis of circadian rhythms, but the influence on mental health is still not well understood.^2^ This is also the case for the impact of seasonal changes on mental health.^24^ More substantial understanding of the many aspects of circadian rhythms and mental health through a variety of different research approaches would address these questions and additionally provide insights into management strategies, feeding into questions 2 and 7.

Despite evidence indicating that several aspects of our circadian biology change over the course of our lives, there is limited evidence as to how these age-related changes influence the relationship between mental health and circadian rhythms.^25 26^ Much remains unclear about the relationship between circadian rhythms and mental health at specific stages of development and times of biological change (such as during adolescence and menopause). This is reflected in questions 1 and 6 of the Top 10 priority questions. To address developmental and biological change, future work may benefit from the inclusion of prospective epidemiological samples and the careful selection and harmonisation of measures at different life stages allowing for comparisons across time-points, as well as specific studies targeting participants in these relevant life stages.

Question 8 of the Top 10 asks whether it would be better for a person’s mental health to follow their own (natural) rhythms or to follow more typical sleep patterns/social patterns. While it is typically assumed that living in line with one’s own body clock is beneficial, further evidence is required and this question acknowledges the wider context of our lives—where school or work start time may be fixed and partners may have a different sleeping pattern to one’s own—factors that must be considered when deciding on the best recommendation for an individual. It is interesting to note that this closely relates to another priority question in the PSP which is concerned with reducing the impact of extreme chronotypes on individuals (question 5). This invites both research and societal debate on whether society in general and employers, in particular, should allow individuals to follow work schedules more suited to their endogenous circadian biology and chronotype to perform better, and live happier and healthier lives.^27^

To maintain a specific focus on mental health and circadian rhythms, the word ‘sleep’ was not used in the PSP remit. Thus, questions about sleep disorders with an aetiology unlikely to be circadian in nature (eg, obstructive sleep apnoea, restless legs syndrome, narcolepsy) were excluded; whereas issues relating to sleep timing were included. The interactions between sleep and sleep disorders and mental health remain important—and would appear to be an appropriate topic for a future, different PSP.

The priorities arising from this exercise necessarily reflect the perspectives of people with lived experience, carers and clinicians, most of whom do not have a specific background in circadian science. It is therefore incumbent on the sleep–circadian research community to work further with stakeholders to interpret and refine these questions into tractable research programmes and translate them into testable interventions. Addressing many of the questions will necessitate the accurate monitoring of the body clock and its environmental determinants in the home environment, which remains a fundamental logistical challenge for the field.^28^

### Limitations

Despite the novel insights that this PSP has provided, this process has several limitations. This is the first PSP to investigate the priorities of circadian rhythms and mental health, which is a wide-reaching area. The PSP may therefore have been less appealing to respondents who had a more specific focus or questions they wanted answered. Anonymous feedback (through social media and respondents speaking to steering group members) suggested that the open-ended questions in the primary survey did not provide enough direction, making it difficult for some respondents to create questions. This potential issue was discussed in the design phase of the primary survey, and it was decided that to avoid influencing respondents, only the most necessary examples and direction would be provided.

The representation of the population captured by the survey was also limited. There was an uneven representation of ethnic and sexual minorities, an under-representation of men and young people, only a small number of clinicians and an over-representation of respondents from Scotland. This is likely a result of the reach of the specific members of the steering group, and the networks that were used to help disseminate the survey. This may also have resulted in respondents with some mental and physical health conditions being represented to a greater extent than others. Due to these limitations, there may have been some influence on the priorities identified. For example, the imbalance of women and men answering the surveys might have been more likely to highlight issues that impact women, such as menopause and menstruation. Although the surveys excluded non-UK residents, the identified priority questions appear universally relevant.

In summary, this study is the first PSP of its kind in the field of mental health and circadian rhythms. The 25 prioritised research questions are the result of a thorough, comprehensive and well-established process in which we collaborated with individuals with lived experience of circadian rhythm disruption and/or mental health difficulties, carers and clinicians. Despite some limitations in the representation of respondents, we recommend consideration of these questions as future priorities for research across the fields of mental health and circadian rhythms. We also recommend that funding bodies consider translating these questions into directed funding calls. The incorporation of lived experience voices should continue when researchers and funding bodies are addressing these priorities.

## Supplementary material

10.1136/bmjment-2025-302101 online supplemental file 1

## Data Availability

Data are available upon reasonable request.
