# Supplementary material for: Research priorities for mental health and circadian science: a priority setting partnership of individuals with lived experience, carers, clinicians and researchers
Source: BMJ Ment Health. 2026 Feb 9;29(1):e302101. doi: 10.1136/bmjment-2025-302101 (PMC12887474; doi:10.1136/bmjment-2025-302101)
Supplement: online supplemental file 1 [file bmjment-29-1-s001.docx]

**Research priorities for mental health and circadian science: a priority setting partnership of individuals with lived experience, carers, clinicians and researchers**

**Supplemental Material**

**Supplemental Figure 1A.** Paper/PDF version of the primary survey, this content matches the online version of the primary survey. **Supplemental Figure 1B.** Paper/PDF version of the ranking survey. The online version of the ranking survey consisted of two stages. Stage 1 has all 63 questions listed in the paper survey and asked participants “Please select all of the questions below that you think are important for research to answer”, stage 2 contained all questions selected by the participant in stage 1 and the participant was asked to select up to 10 that were most important to them.

**Supplemental Figure 1A.**

**
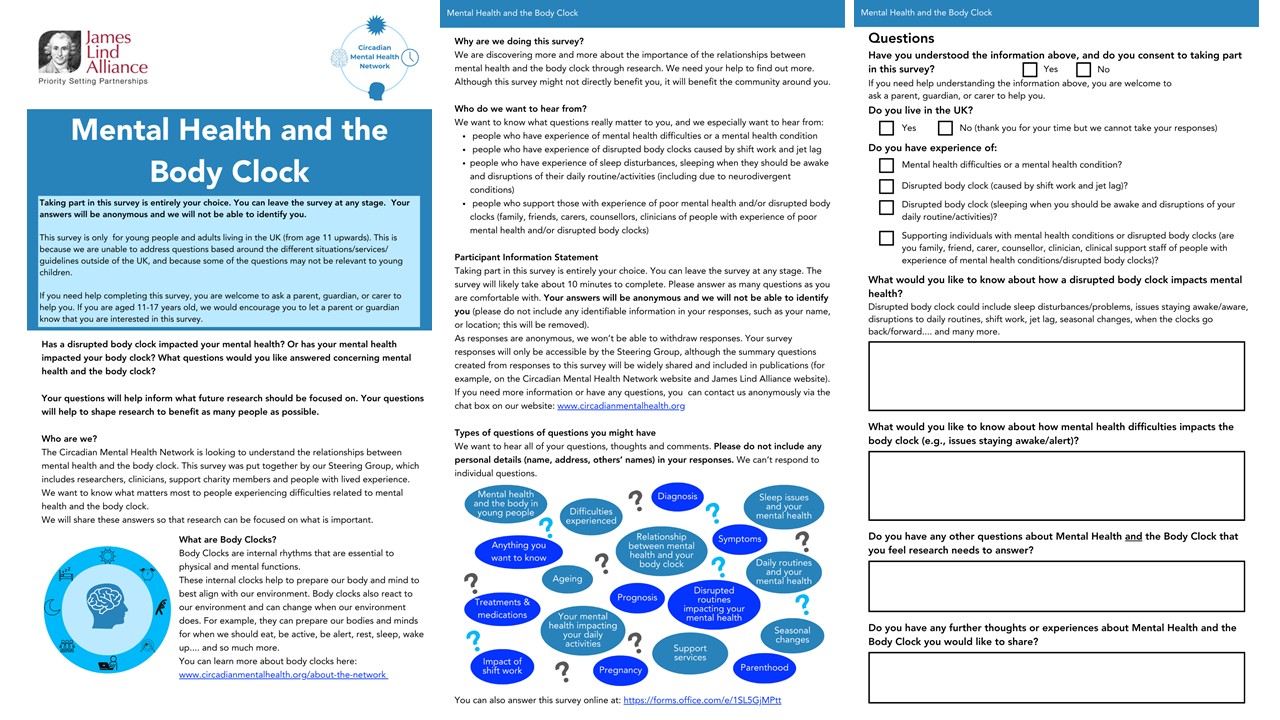
**

**
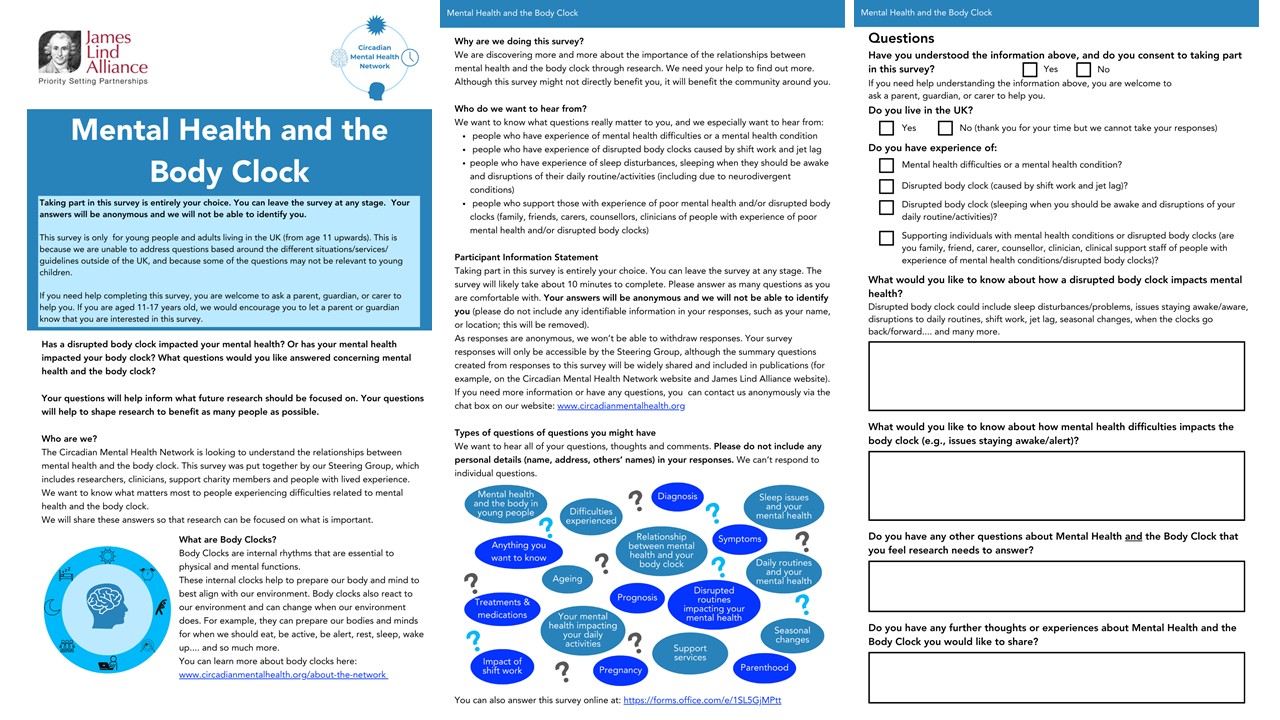
**

**
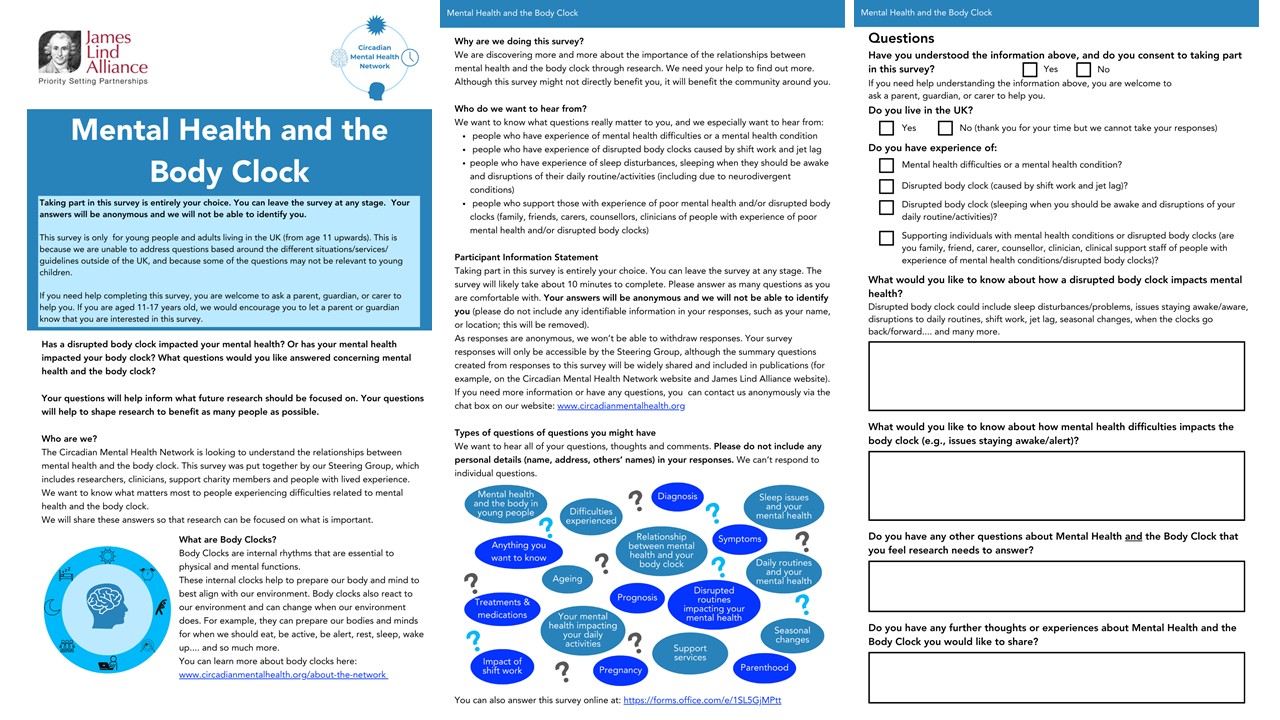
**

**
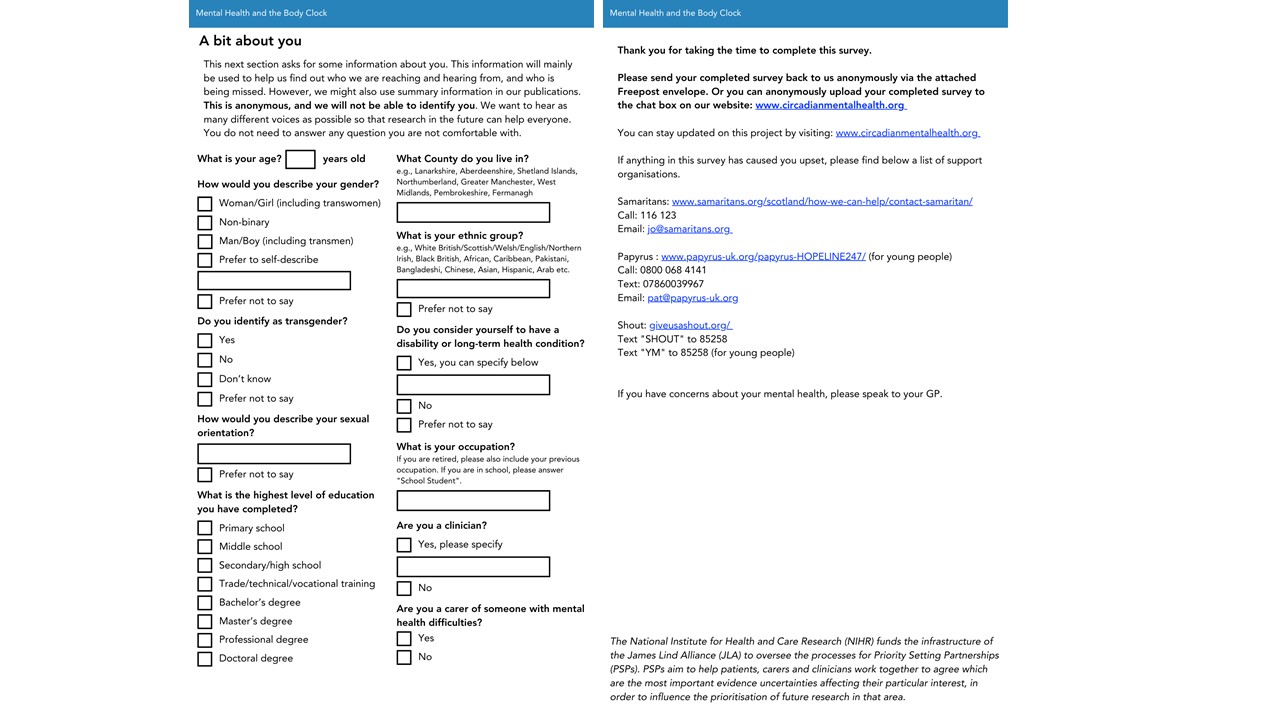
**

**
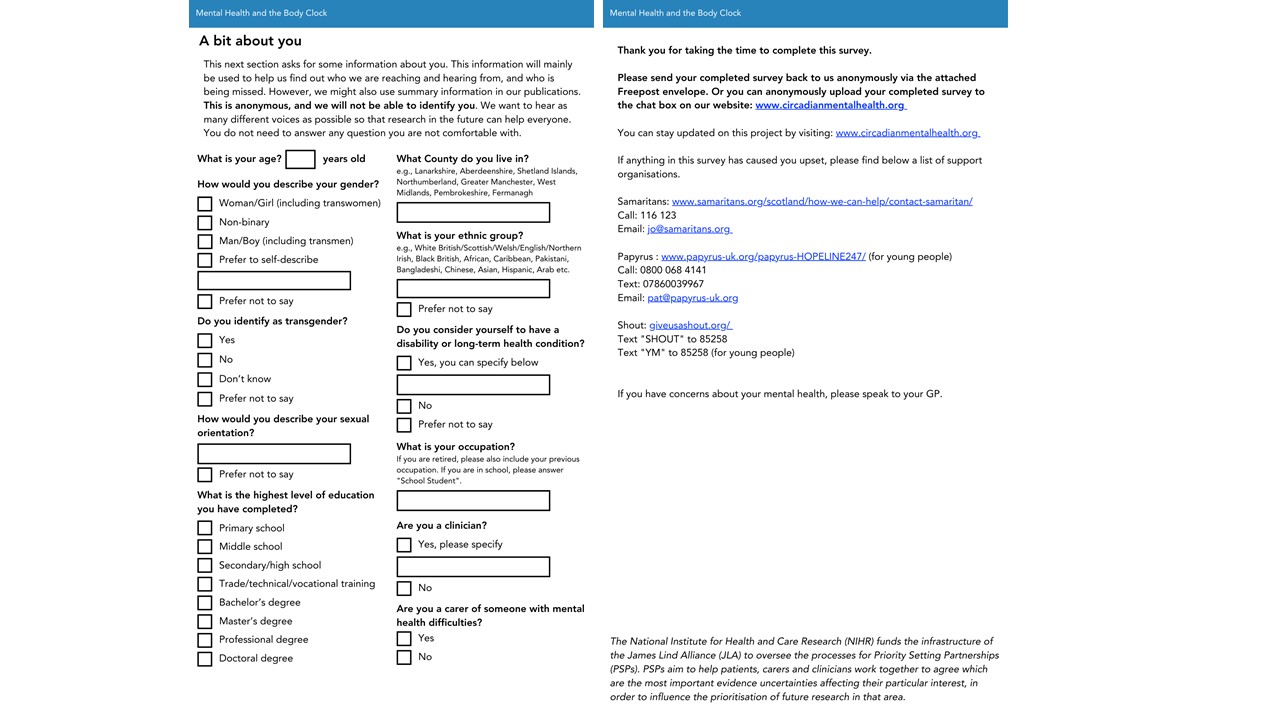

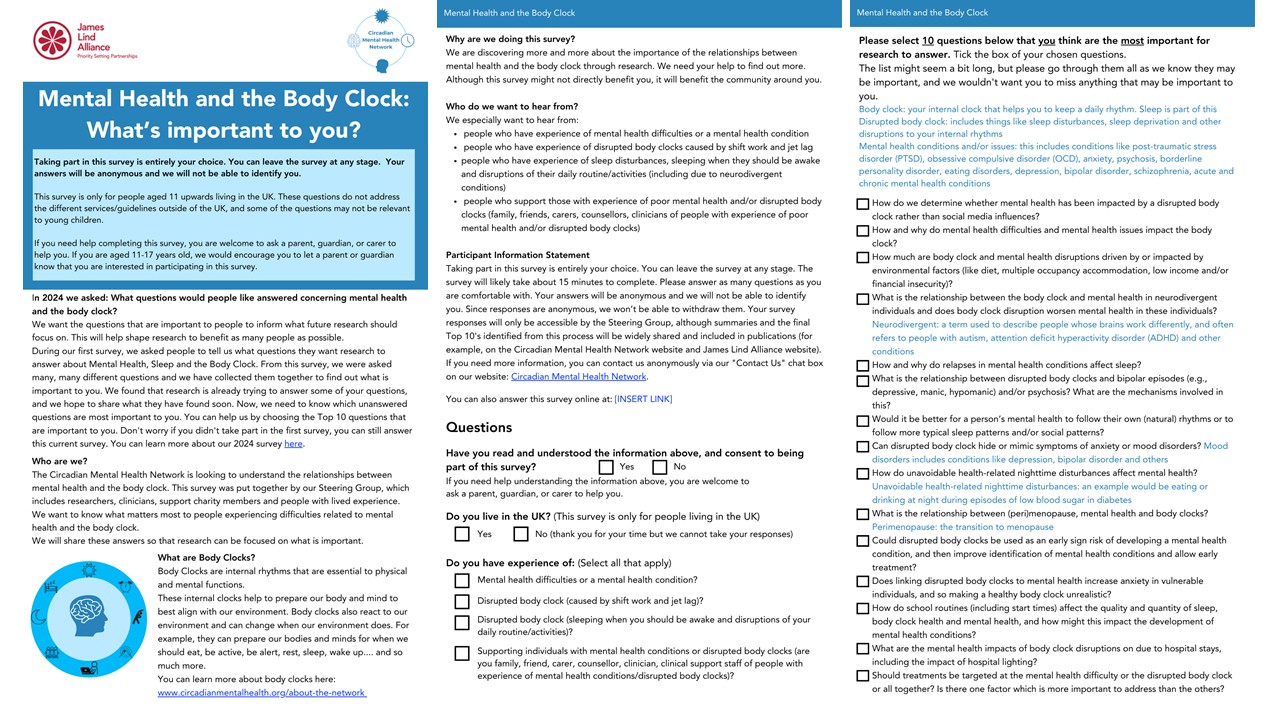
Supplemental Figure 1B.**

**
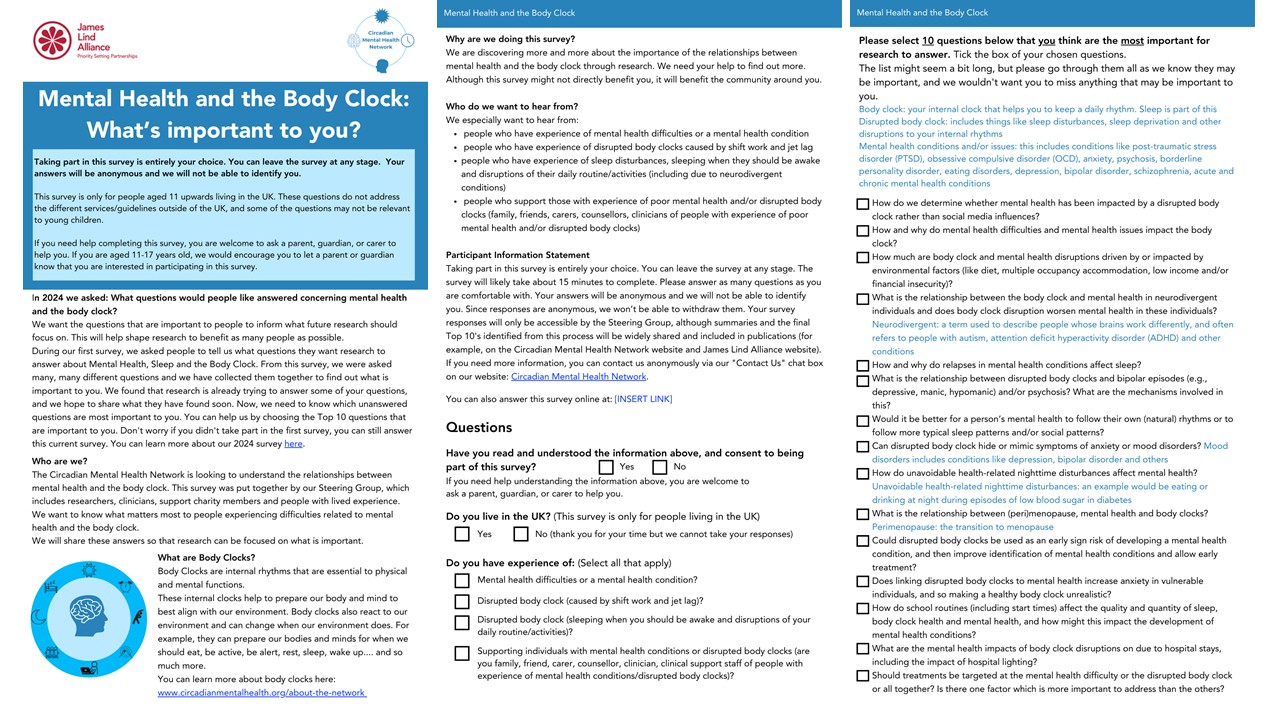
**

**
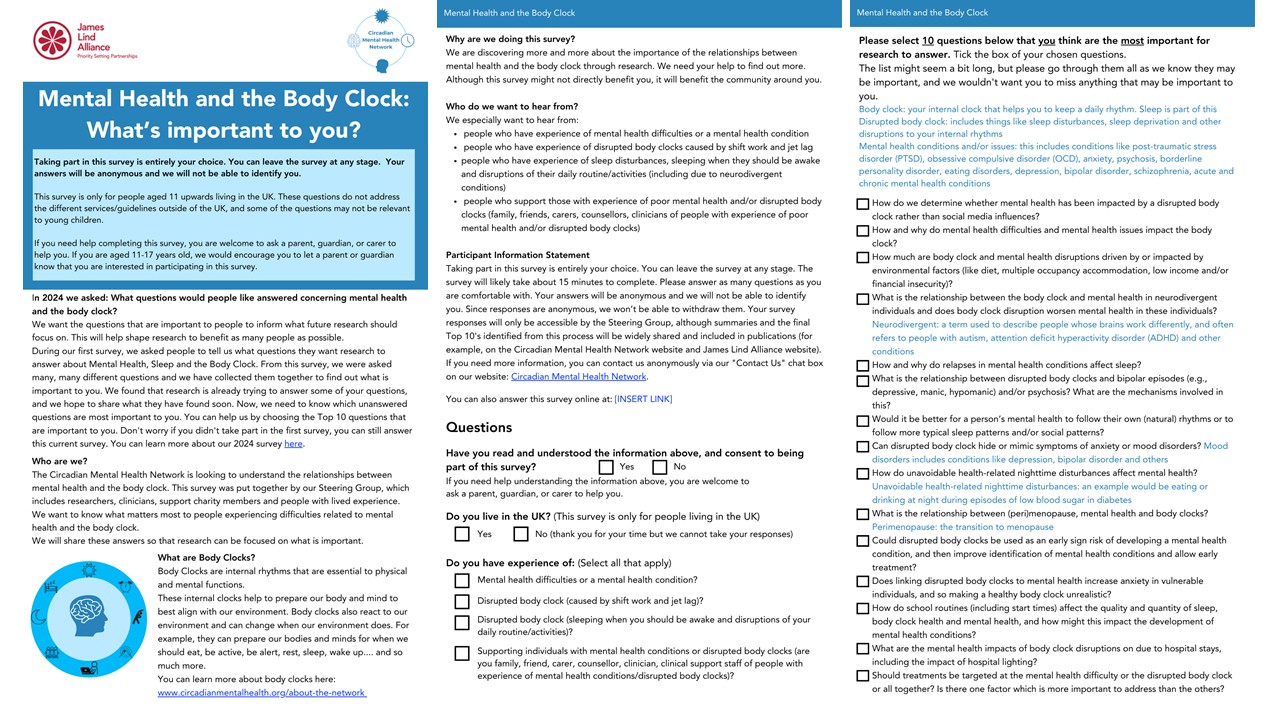
**

**
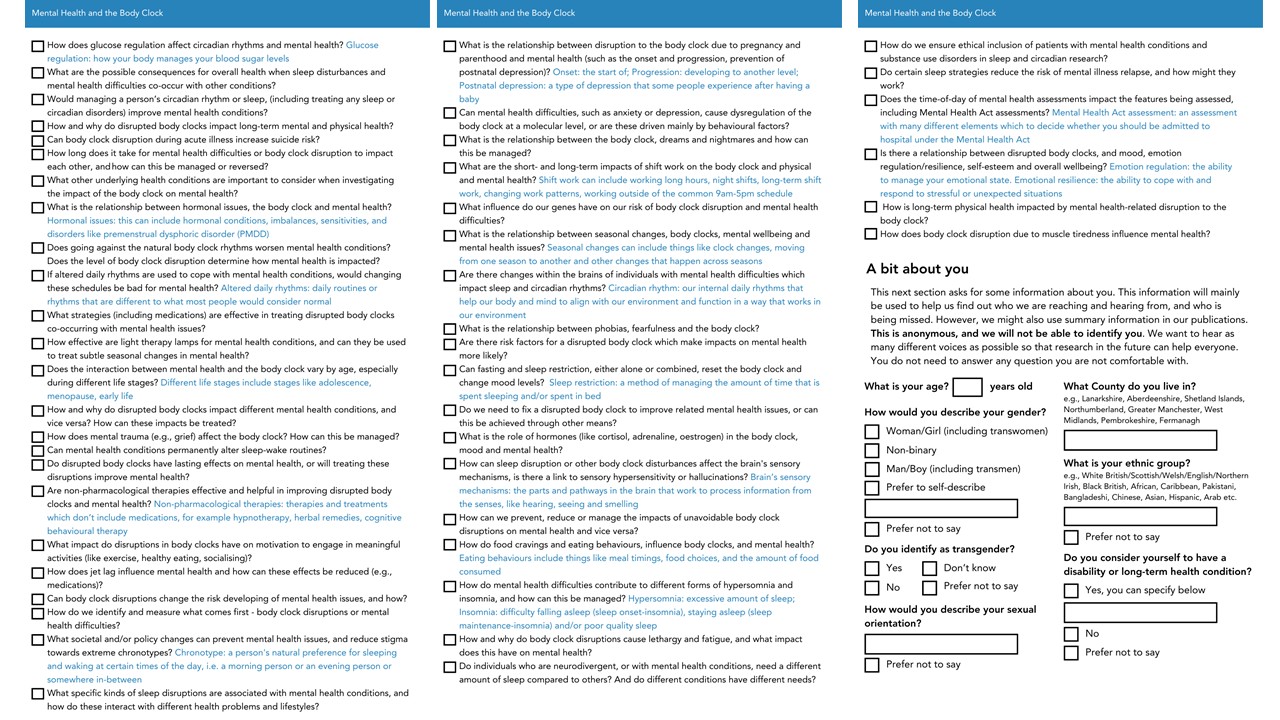
**

**
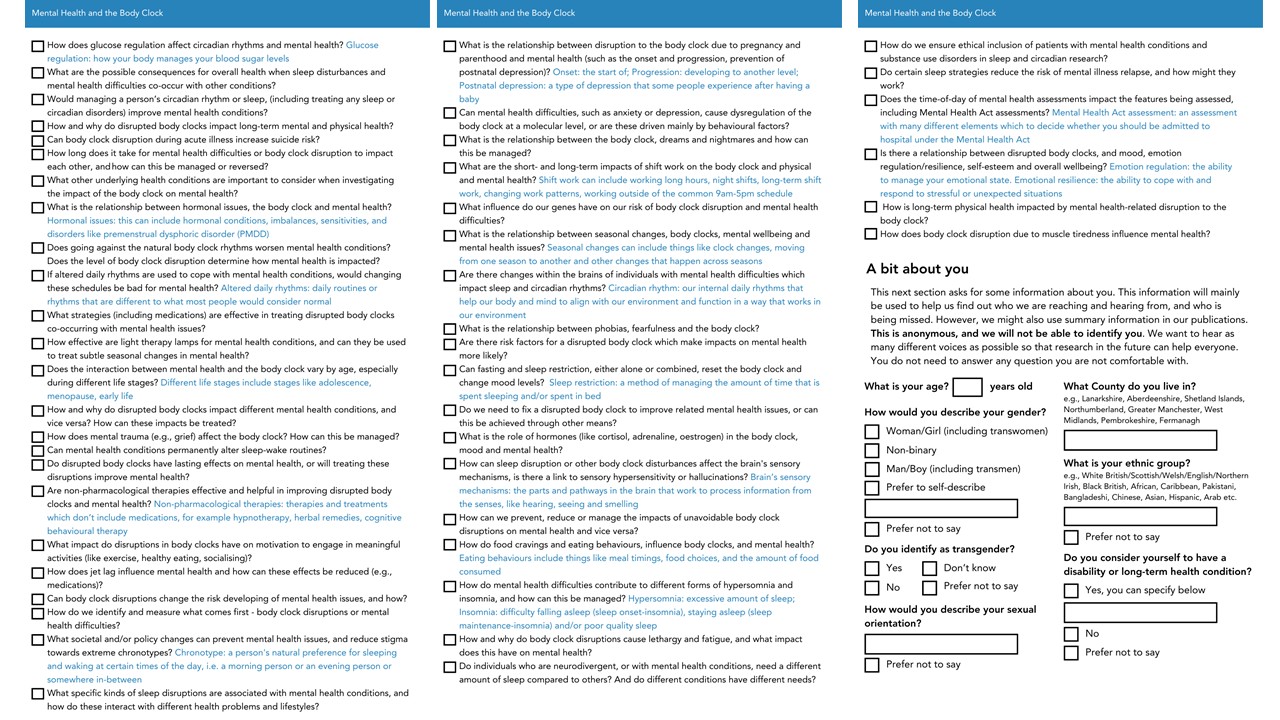
**

**
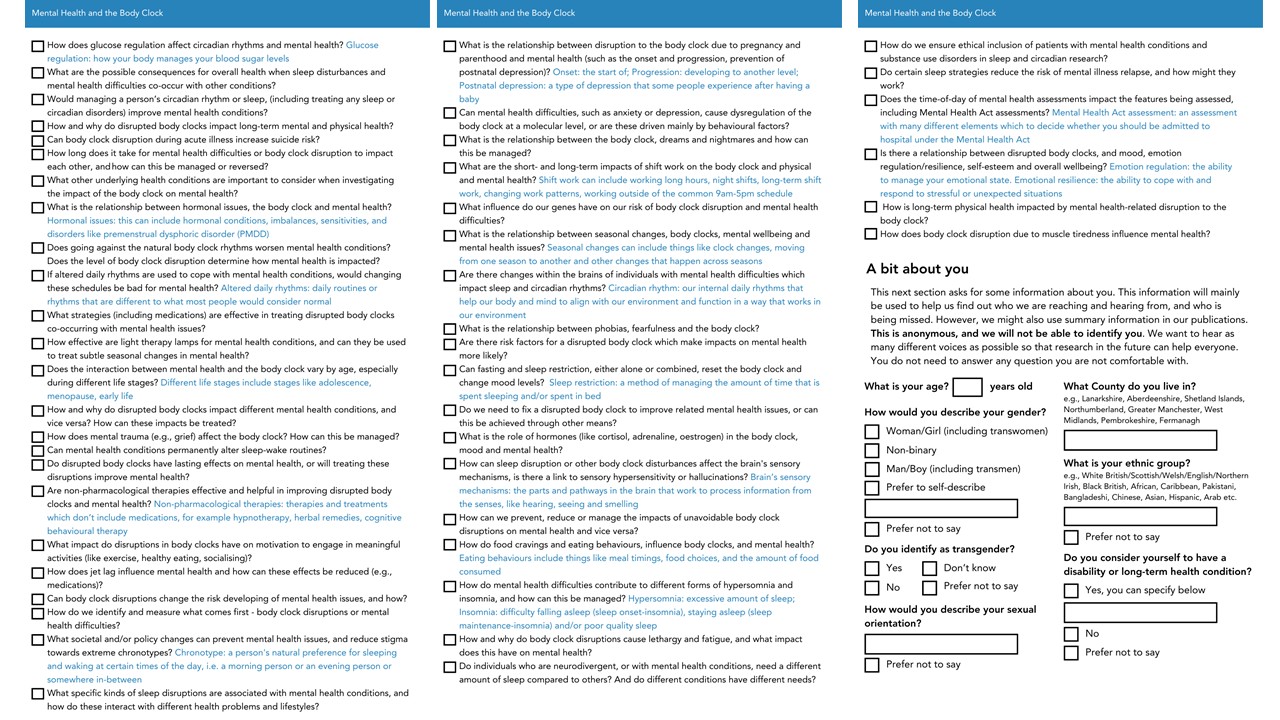
**

**Supplemental Table 1.** Themes identified through primary survey responses.

| **Themes identified from primary survey responses** | |
| --- | --- |
| Age related | Mental Health Impacts |
| Alertness | Mental Health problems/issues |
| Anxiety | Mental Health treatment |
| Alternative therapies | Mood |
| Body clock health | Neurodivergence/neurodevelopmental |
| Body clock impacts | Negative thoughts |
| Body clock treatment | Other conditions |
| Bipolar Disorder | Obsessive Compulsive Disorder |
| Brain impact | Parenthood |
| Brain | Perception |
| Cells/Tissues/Genetic | Physical health |
| Children/School | Poverty |
| Chronotype | Pregnancy |
| Cognition | Problems caused |
| Connection/relationship | Psychosis |
| Depression | Post-traumatic Stress Disorder |
| Dreams and nightmares | Risk factors |
| Diet | Rhythm reset |
| Eating Disorders | Rumination |
| Emotions | Suicide risk |
| Environment | Schizophrenia |
| Exercise | Sleep hygiene |
| Gender | Sleep |
| Hormones | Social media |
| Hospital/inpatient | Seasonality |
| Health (overall/physical) | Sleep Quality |
| Hypersomnia | Stress |
| Insomnia | Substances (drugs/alcohol) |
| Jet lag | Support |
| Lifestyle changes | Shift work |
| Light | Tiredness |
| Mania | Trauma |
| Medication | Tests |
| Menopause | Waking |
| Mental Health Conditions |  |

**Supplemental Table 2.** Top 25 priorities for research in mental health and circadian rhythms

| **Final ranking in Top 25** | **Question** | **Ranking from second survey by** | | |
| --- | --- | --- | --- | --- |
|  |  | **People with lived experience** | **Carers** | **Clinicians or clinical support workers** |
| **1** | Does the interaction between mental health and the body clock vary by age, especially during different life stages? | 15^th^ | 1^st^ | 2^nd^ |
| **2** | What strategies (including medications) are effective in treating disrupted body clocks co-occurring with mental health issues? | 13^th^ | 5^th^ | 6^th^ |
| **3** | What is the relationship between the body clock and mental health in neurodivergent individuals and does body clock disruption worsen mental health in these individuals? | 8^th^ | 3^rd^ | 5^th^ |
| **4** | What is the relationship between a disrupted body clock and bipolar disorder, or between a disrupted body clock and psychosis? What are the mechanisms involved in this? | 11^th^ | 8^th^ | 4^th^ |
| **5** | What societal and/or policy changes can help prevent mental health issues for, and reduce stigma towards, extreme chronotypes? | 13^th^ | 8^th^ | 8^th^ |
| **6** | What is the relationship between (peri)menopause, mental health and body clocks? | 2^nd^ | 2^nd^ | 1^st^ |
| **7** | How does mental trauma (e.g., grief) affect the body clock? How can this be managed? | 9^th^ | 7^th^ | 8^th^ |
| **8** | Would it be better for a person’s mental health to follow their own (natural) rhythms or to follow more typical sleep patterns and/or social patterns? | 4^th^ | 9^th^ | 6^th^ |
| **9** | What is the relationship between seasonal changes, body clocks, mental wellbeing and mental health issues? | 6^th^ | 4^th^ | 7^th^ |
| **10** | Can mental health difficulties, such as anxiety or depression, cause disruption of the body clock at a molecular level, or are these driven mainly by behavioural factors? | 13^th^ | 6^th^ | 4^th^ |
| **11** | What is the relationship between hormonal issues (including imbalances, sensitivities and conditions, such as premenstrual dysphoric disorder (PMDD)), the body clock and mental health? | 12^th^ | 5^th^ | 6^th^ |
| **12** | Are there changes within the brains of individuals with mental health difficulties which impact sleep and circadian rhythms? | 13^th^ | 6^th^ | 7^th^ |
| **13** | What is the relationship between disruption to the body clock due to pregnancy and parenthood and mental health (such as the onset, progression, and prevention of postnatal depression)? | 2^nd^ | 3^rd^ | 1^st^ |
| **14** | What are the short- and long-term impacts of shift work on the body clock and physical and mental health? | 17^th^ | 4^th^ | 5^th^ |
| **15** | Is there a relationship between disrupted body clocks, and mood, emotion regulation/resilience, self-esteem and overall wellbeing? | 8^th^ | 4^th^ | 9^th^ |
| **16** | How effective are light therapy lamps for mental health conditions, and can they be used to treat subtle seasonal changes in mental health? | 14^th^ | 7^th^ | 10^th^ |
| **17** | What are the mental health impacts of body clock disruptions due to hospital stays, including the impact of hospital lighting? | 14^th^ | 10^th^ | 5^th^ |
| **18** | How does glucose regulation affect circadian rhythms and mental health? | 7^th^ | 6^th^ | 8^th^ |
| **19** | How do food cravings and eating behaviours, influence body clocks, and mental health? | 9^th^ | 2^nd^ | 9^th^ |
| **20** | Are non-pharmacological therapies (such as hypnotherapy, herbal remedies) effective and helpful in improving disrupted body clocks and mental health? | 5^th^ | 2^nd^ | 5^th^ |
| **21** | What is the role of hormones (like cortisol, adrenaline, oestrogen) in the body clock, mood and mental health? | 1^st^ | 2^nd^ | 3^rd^ |
| **22** | How do school routines (including start times) affect the quality and quantity of sleep, body clock health and mental health, and how might this impact the development of mental health conditions? | 17^th^ | 5^th^ | 6^th^ |
| **23** | Would managing a person’s circadian rhythm or sleep, (including treating any sleep or circadian disorders) improve mental health conditions? | 10^th^ | 5^th^ | 5^th^ |
| **24** | Do individuals who are neurodivergent, or individuals with mental health conditions, need a different amount of sleep compared to others? And do different conditions have different needs? | 3^rd^ | 1^st^ | 7^th^ |
| **25** | Does the time-of-day of mental health assessments impact the features being assessed, including Mental Health Act assessments? | 21^st^ | 8^th^ | 5^th^ |
